# Mediating Effect of Body Mass Index and Dyslipidemia on the Relation of Uric Acid and Type 2 Diabetes: Results From China Health and Retirement Longitudinal Study

**DOI:** 10.3389/fpubh.2021.823739

**Published:** 2022-01-28

**Authors:** Fang Cheng, Yanzhi Li, Han Zheng, Lu Tian, Hongying Jia

**Affiliations:** ^1^Department of Epidemiology and Health Statistics, School of Public Health, Cheeloo College of Medicine, Shandong University, Jinan, China; ^2^Center of Evidence-Based Medicine, Institute of Medical Sciences, The Second Hospital, Cheeloo College of Medicine, Shandong University, Jinan, China

**Keywords:** diabetes, BMI, uric acid, dyslipidemia, mediation effect

## Abstract

**Objective:**

This study assessed temporal relationships of serum uric acid (SUA) with blood glucose and determine the mediating effects of body mass index (BMI) and dyslipidemia on the relation of SUA and risk of type 2 diabetes.

**Methods:**

Participants aged ≥ 45 years were participated in 2011 and followed up until 2015. Cox proportional hazards regression with a robust variance estimator was performed to explore the association of SUA with the risk of diabetes, and crosslagged path analysis was introduced to examine the temporal relationships between SUA and blood glucose. A mediation analysis was finally used to identify the mediating effect of BMI and dyslipidemia on the relation of SUA and the future risk of diabetes.

**Results:**

A total of 9,020 participants were included with an average age of 58.59 years at baseline in 2011, and 53.6% of them were women. Linear dose–response relationship was identified by restricted spline cubic analysis between baseline SUA and follow-up blood glucose (the non-linear trend for fasting plasma glucose (FPG): β_2_ = −0.71, *p* = 0.52; for HbA1c: β_2_ = 0.05, *p* = 0.07; for risk of diabetes: β_2_ = 0.12, *p* = 0.39). Additionally, compared with the lowest quartiles of SUA, the adjusted risk ratios of diabetes were 1.00 (95% *CI*: 0.82–1.23), 1.08 (95% *CI*: 0.89–1.31), and 1.37 (95% *CI*: 1.11–1.96) for quartile 2–4 (*p*-trend < 0.01), respectively. Further additional adjustments for BMI or dyslipidemia, these ratios were not statistically significant. In addition, a unidirectional relationship from baseline SUA to follow-up FPG (ρ_1_ = 0.24, *p* = 0.03) was further confirmed using crosslagged path analysis. After stratifying by genders, the above results were only significant in the women subgroup, and we thus conducted a mediation analysis in women and found that the BMI and dyslipidemia partially mediated the effect of SUA on diabetes with a 23.05 and 18.82% mediating effect, respectively.

**Conclusions:**

These findings provide strong evidence that hyperuricemia preceded diabetes, and the effect of baseline SUA on follow-up type 2 diabetes was more pronounced among middle-aged and elderly Chinese women, especially in postmenopausal women, and this effect is partly mediated by BMI and dyslipidemia at baseline.

## Introduction

Type 2 diabetes is a metabolic disease characterized by insulin resistance, which affected over 463 million people in 2019, and this number is expected to increase to 578 million in 2,030 and 700 million in 2045 (1). Identifying all potential controllable risk factors for the incidence and development of diabetes is essential for its early screening and prevention.

As the main component of metabolic syndrome, diabetes, hyperglycemia, and hyperlipidemia interconnect and influence each other, forming a complex framework of chronic diseases (2). The link between hyperuricemia and diabetes has been well documented in the previous studies (3–6), some of them demonstrated that for every 1 mg/dL increase in serum uric acid (SUA) concentration, the risks of type 2 diabetes were increased by 6–11% (6), and these studies also showed differences between genders. Meanwhile, the epidemiological and clinical evidence supports a strong significant positive association between SUA and obesity in the adult population of China, Japan, India, Pakistan, and Iraq (7, 8). About 44% of diabetes cases are overweight or obese (9), and adults with body mass index (BMI) > 35 kg/m^2^ are 20 times as likely to develop type 2 diabetes than those with a BMI between 18.5 and 24.9 kg/m^2^. Also, some researchers reported that the relation of SUA and diabetes is largely decreased or eliminated when additional adjusting BMI (10, 11). The possible mechanism is that hyperuricemia can cause obesity by accelerating liver and peripheral fat production (12). In addition, dyslipidemia is a common comorbidity in patients with diabetes (13), and over 70% of them have one or more lipid abnormalities. Low levels of high-density lipoprotein cholesterol (HDL-C) are often associated with elevated triglyceride levels, the most prevalent form of dyslipidemia in patients with diabetes (14). Studies illustrated that SUA can inhibit the synthesis of adiponectin in adipocytes by reducing the production of nitric oxide in arterial endothelial cells, disrupting the tricarboxylic acid cycle and the oxidation of fatty acid β, and finally promoting the oxidative activity of cells (15).

Besides, available data suggest that uric acid is not necessarily an antioxidant and, depending on the chemical milieu, may become a prooxidant (16, 17). This partly explained the U- or L-shaped association between SUA and blood glucose reported in previous cross-sectional studies (18, 19). What is more, Rodriguez and his colleagues identified that individuals with prediabetes are at a higher risk of developing gout, but once they develop diabetes, their risk drops to a lower level than that of non-diabetic individuals, and diabetes may reduce the future risk of gout through the uricosuric effect of glycosuria or the impaired inflammatory response (20).

To clarify the complex interaction between metabolic syndrome components and formulate reasonable diabetes control measurements, this study collected data from a nationally representative database, the China Health and Retirement Longitudinal Study (CAHRLS), to initially explore whether the uric acid level is independently related to the future risk of diabetes and then introduced a restricted cubic spline function to identify whether there is a non-linear relationship between baseline uric acid and follow-up blood glucose; further used crosslagged path analysis to determine the temporal relation of blood uric acid and blood glucose. Once the temporal relationship is established, we would investigate the mediating effect of BMI and dyslipidemia on the relation of uric acid and risk of diabetes.

## Materials and Methods

### Study Population

The China Health and Retirement Longitudinal Study (CHARLS) takes the mainland of China as the sampling frame, the community (in the city) or village (in urban) as the sampling unit, and uses probability proportionate to size sampling (PPS) as the sampling technology, to investigate the information on health and retirement of middle-aged and elderly people aged 45 or over, with no upper age limit (21). The CHARLS data can be freely downloaded from the official website (http://charls.pku.edu.cn/index/zh-cn.html). So far, CHARLS yields four waves of data in 2011, 2013, 2015, and 2018 and two blood test data in 2011 and 2015. Information about demographics, biomedical measurements, socioeconomic status, and self-reported health status and functioning was measured by trained health workers (21), and all participants were asked to take venous blood on fasting overnight (22). The CHARLS is a large-scale interdisciplinary research project sponsored by the National Development Institute of Peking University. Ethical approval for all the CHARLS waves, therefore, was granted from the Institutional Review Board (IRB) at Peking University, including anthropometrics (IRB00001052-11015) and biomarker collection (IRB00001052-11014).

**Figure 1:**
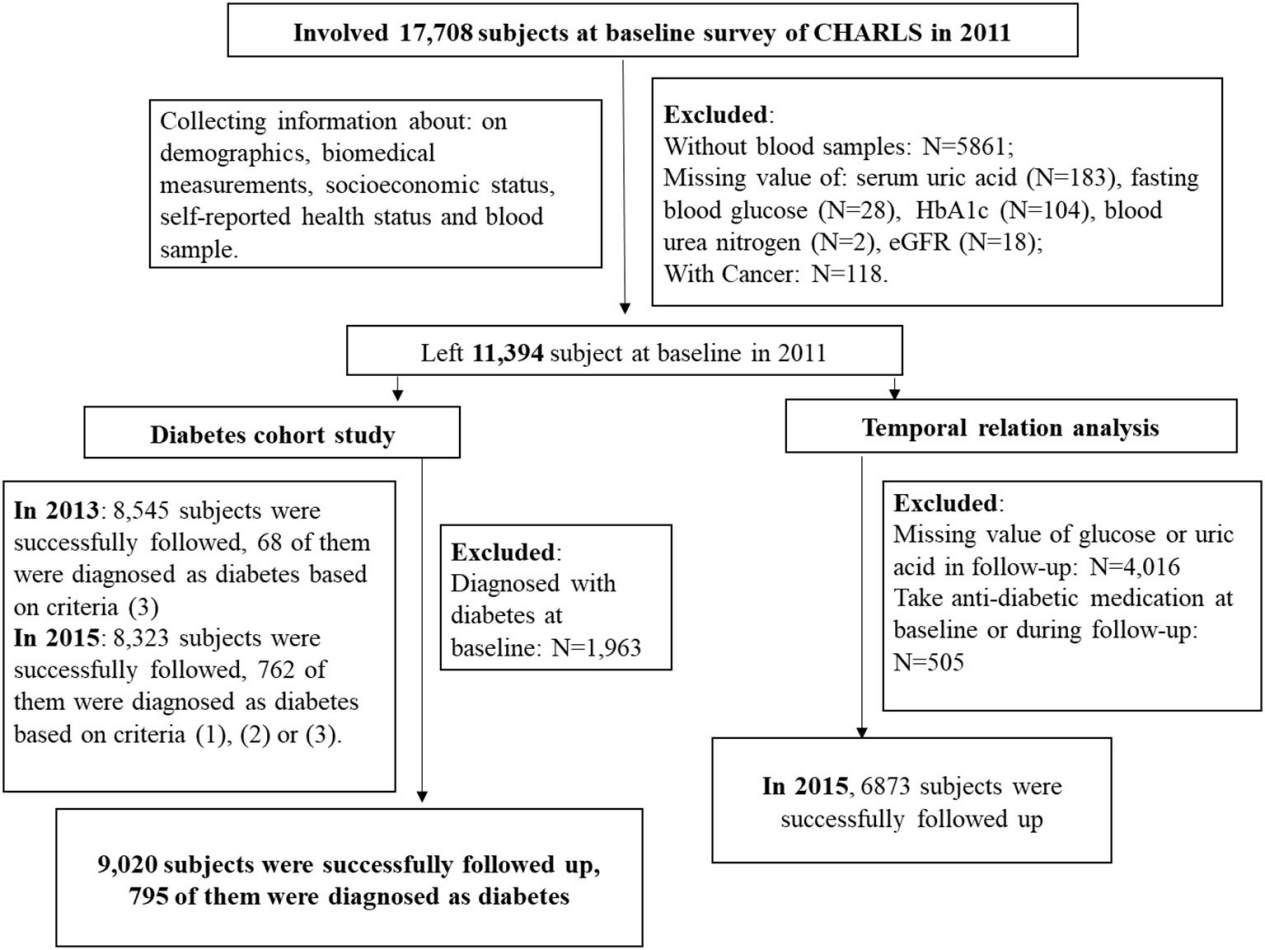
Flow diagram of patients included in this study.

In this study, at baseline (2011), a total number of 17,708 respondents completed a face-to-face computer-assisted personal interview, and the exclusion criteria were as follows: (1) without blood samples; (2) missing value of SUA, fasting blood glucose (FPG), hemoglobin A1c (HbA1c), blood urea nitrogen (BUN), estimated glomerular filtration rate (eGFR); (3) with cancer; and (4) diagnosed with type 2 diabetes. A number of 9,431 participants were remained at baseline after exclusion criteria. In 2013 and 2015, 8,545 and 8,323 subjects were successfully followed, and 68 and 762 of them were diagnosed with diabetes, respectively. A number of 9,020 participants were finally followed up, and 795 of them were diagnosed with type 2 diabetes. More details are shown in Figure 1.

### Demographic, Anthropometric, and Biochemical Parameters

According to the common classification in the existing literature, we divided the education level into the following four levels, including no formal education or illiterate, elementary or below, middle school, and high school or above. Based on the setting of the questionnaire, we simply divided marital status married or living with a partner, vs. others. Blood pressure was measured using an Omron HEM-7200 sphygmomanometer in the sitting position, three consecutive measurements were taken using the standard method, 45 s apart, and the average of the three results was taken as the final blood pressure. Hypertension means systolic blood pressure ≥ 140 mmHg or diastolic blood pressure ≥ 90 mmHg, and we also classified people into the hypertension group who had been diagnosed with hypertension by their doctor or currently using antihypertensive medication. A higher level of high-sensitivity C-reactive protein (hs-CRP) over 3 mg/dL indicates inflammation in the body (23). The estimated eGFR was calculated using the Chronic Kidney Disease Epidemiology Collaboration equation (24).

### Definition of Primary Variables

Participants who meet one of the following criteria are considered to have type 2 diabetes: (1) FPG ≥ 7.0 mmol/L (126 mg/dL); (2) random plasma glucose (without overnight fasting) ≥ 11.1 mmol/L (200 mg/dL); (3) HbA1c ≥ 48 mmol/mol (6.5%), (4) self-reported physician-diagnosed diabetes; and (5) currently taking antidiabetic medication.

The definition of hyperuricemia is different for men and women, for men with SUA ≥ 420 μmol/L, and for women with SUA ≥ 360 μmol/L (25). We further divided participants into four groups using SUA gender-specific quartiles.

Once participants met one of the following criteria, they were diagnosed with dyslipidemia: (1) total cholesterol (TC) ≥ 240 mg/dl, (2) high-density lipoprotein cholesterol (HDL-C) <40 mg/dl, (3) low-density lipoprotein cholesterol (LDL-C) > 160 mg/dl, and (4) triglycerides (TG) ≥ 200 mg/dl (26).

Body mass index was calculated as dividing weight (kilogram) by height (meter) squared, and they were further divided into four categories (27), as follows: underweight (BMI <18.5 kg/m^2^), normal weight (BMI <24 kg/m^2^), overweight (BMI <28 kg/m^2^), and obese (BMI ≥ 28 kg/m^2^).

### Statistical Analysis

Statistical analysis was conducted by STATA version 16.0 (StataCorp, College Station, TX, USA) and R 4.0.3 (R Project for Statistical Computing), and a *p*-value < 0.05 (2-tailed) was considered statistically significant. To make this study represent the overall level of middle-aged and elderly people in China, we took blood weight published in the CHARLS database in 2011 as the initial weight, further introducing the jackknife method to conduct weighted analysis based on the PPS sampling design. Baseline characteristics of study participants were reported by percentages for categorical variables or mean (standard error, SEM) for continuous variables. Groups were compared with one-way analysis of variance or the Kruskal–Wallis test for continuous variables, and Cochran–Mantel–Haenszel chi-square test for categorical variables.

Since new cases of diabetes were investigated at the 2013 or 2015 follow-up surveys, we were unable to estimate person-year accurately, in addition to the high incidence of diabetes in middle-aged and elderly people, prevented us from using odds ratios (*OR*) calculated by logistic regression to estimate relative risk (*RR*), since the use of *OR* instead of *RR* is artificially appropriate for rare events, Instead, we introduced the Cox proportional hazards regression with a robust variance estimator to estimate the *RR*, we set the follow-up time to 1, and we used the Breslow method to break ties (28).

In addition, our previous study has confirmed an L-shape association between SUA and blood glucose at the same measurement point (19), but we did not know whether the relation of baseline SUA and the risk of diabetes during follow-up is linear. So, we introduced restricted cubic spline models to examine the dose–response association of SUA (continuous) with follow-up blood glucose (continuous). If the above dose–response relationship was linear, we further conducted Cox proportional hazards regression to evaluate the association of SUA quartiles or continuous SUA levels (per 100 μmol/L elevations) or hyperuricemia (yes/no) with the risk of diabetes.

Further, the longitudinal changes of uric acid and blood glucose measured at two-time points are typically a crosslagged panel design (29). A regression residual analysis was initially used to identify the baseline and follow-up uric acid and blood glucose after adjusting all potential confounding factors, and then, values of adjusted residuals were standardized by Z-transformation (mean = 0, standard deviation = 1). The crosslagged path coefficients (ρ_1_ and ρ_2_) were estimated simultaneously based on the correlation matrix using the maximum likelihood method in R 4.0.3 (Package: “lavaan”). The validity of model fitting was indicated by the root mean square residual (RMR) and comparative fitness index (CFI) (30), and RMR <0.05 and CFI > 0.90 indicate good fit to the observed data.

Finally, a Karlson–Holm–Breen (KHB) (31) method was constructed to examine the mediation effect of baseline BMI and dyslipidemia on the association between baseline SUA and follow-up risk of diabetes.

## Results

### The Characteristics Regarding the Study Variables

Baseline demographic and clinical characteristics of the study population are given in Table 1. A total of 9,020 individuals (4,185 men and 4,835 women) were included in this study. At baseline, the mean BMI was 23.31 kg/m^2^, the FPG level was 99.63 mg/dl, the uric acid level was 270.05 μmol/L, the HbA1c level was 5.08%, and the prevalence of dyslipidemia was nearly 39.55%. After 4 years of follow-up, BMI increased by 0.4 units and SUA levels increased by 20 umol/L. A total of 795 participants were diagnosed with new-onset diabetes, with a cumulative 4-year incidence of 8.81%. Compared with women, men were more likely to be current smokers, regular drinkers, with higher hs-CRP, BUN, and SUA levels at baseline. In contrast, never smoking, never drinking, being illiterate, with higher BMI, and eGFR level were more common in women.

**Table 1:** Weighted characteristics of the study participants according to different gender.

	**All**	**Men**	**Women**	***p-*value**
**Baseline (2011)**				
*n*	9,020	4,185	4,835	
Rural (%)	54.21	54.74	53.71	0.41^†^
Married or living with a partner (%)	82.74	86.80	78.77	<0.001^†^
**Smoking status (%)**				<0.001^†^
Never smoker	62.82	28.44	93.08	
Former smoker	9.09	17.48	1.71	
Current smoker	28.08	54.08	5.21	
**Alcohol consumption (%)**				<0.001^†^
Never drinking	58.71	59.31	83.90	
Former drinkers	7.20	7.26	3.79	
Occasional drinkers	8.61	8.69	5.64	
Regular drinkers	25.47	24.74	6.67	
**Education levels (%)**				<0.001^†^
No formal education	24.73	11.96	36.38	
Elementary or below	39.51	43.62	35.76	
Middle school	22.49	27.98	17.48	
High school or above	13.27	16.44	10.38	
Dyslipidemia (%)	39.55	40.37	38.79	0.38^†^
High hs-CRP (%)	17.50	19.58	15.60	<0.01^†^
Hypertension (%)	41.84	40.86	42.72	0.18^†^
Age (years)	58.59 (0.21)	59.16 (0.27)	58.07 (0.24)	<0.001^§^
BMI (kg/m^2^)	23.31 (0.07)	22.86 (0.08)	23.74 (0.06)	<0.001^§^
Systolic (mmHg)	130.83 (0.36)	130.88 (0.43)	130.77 (0.36)	0.46^‡^
Diastolic (mmHg)	75.64 (0.22)	76.13 (0.31)	75.20 (0.24)	<0.001^§^
WC (cm)	83.81 (0.24)	83.63 (0.31)	83.97 (0.23)	<0.01^§^
BUN (mg/dl)	15.65 (0.09)	16.41 (0.10)	14.96 (0.10)	<0.001^§^
eGFR (ml/min/1.73 m^2^)	85.56 (0.23)	84.13 (0.24)	86.89 (0.28)	<0.001^§^
HDL-C (mg/dl)	50.75 (0.33)	49.95 (0.33)	51.49 (0.26)	<0.001^§^
LDL-C (mg/dl)	115.54 (0.56)	112.80 (0.64)	118.04 (0.65)	<0.001^‡^
T-cho (mg/dl)	189.77 (0.69)	185.75 (0.69)	193.44 (0.72)	<0.001^§^
TG (mg/dl)	123.79 (1.74)	119.68 (2.23)	127.54 (1.85)	<0.001^§^
SUA (μmol/L)	270.05 (1.27)	300.87 (1.81)	241.85 (1.30)	<0.001^§^
FPG (mg/dl)	99.63 (0.23)	99.64 (0.30)	99.62 (0.25)	0.65^§^
HbA1c (%)	5.08 (0.02)	5.08 (0.01)	5.07 (0.01)	0.55^‡^
**Follow-up (2015)**				
BMI (kg/m^2^)	23.76 (0.05)	23.21 (0.06)	24.26 (0.06)	<0.001^§^
SUA (μmol/L)	298.11 (1.47)	331.94 (2.10)	268.57 (1.46)	<0.001^§^
FPG (mg/dl)	98.13 (0.35)	98.73 (0.50)	97.61 (0.44)	0.83^§^
HbA1c (%)	5.81 (0.01)	5.78 (0.01)	5.84 (0.01)	<0.001^§^
Diabetes (%)	8.81	8.48	9.10	0.30^†^

*Unless indicated otherwise, data are given as the means (SEM) or as percentages.*

*Cochran –Mantel–Haenszel chi-square test.*

*^‡^One-way analysis of variance (ANOVA).*

*^§^The Kruskal–Wallis test*.

### Dose–Response Relation of Baseline Uric Acid and Incidence Diabetes

**Figure 2:**
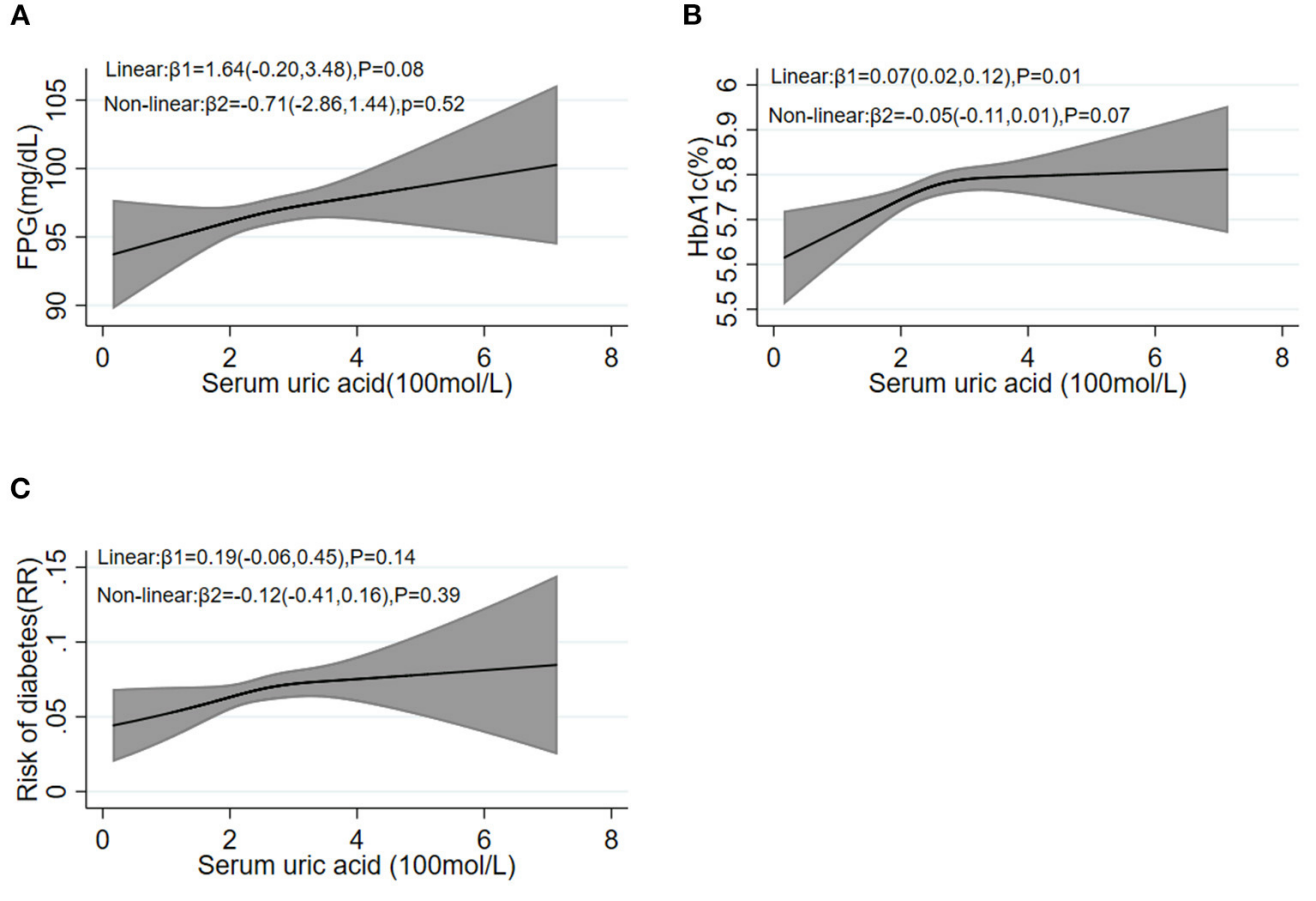
Restricted cubic spline analysis between baseline SUA levels and follow-up FPG **(A)**, HbA1c **(B)**, and the risk of diabetes **(C)**. FPG, fasting plasma glucose; HbA1c, hemoglobin A1c.

As shown in Figure 2, the β_2_ coefficient of non-linear trend calculated by restricted cubic spline was not significant, suggesting a linear dose–response association of SUA with blood glucose [FPG (Figure 2A) and HbA1c (Figure 2B) as continuous variables, diabetes (Figure 2C) as a dichotomous variable, respectively].

The linear coefficients between SUA and diabetes are shown in Table 2. In the total samples, compared with the lowest sex-specific quartile of SUA levels, individuals in the higher quartiles of SUA had a higher incident risk of diabetes. The *RR*s of incident diabetes were 1.00 (95% *CI*: 0.82–1.23), 1.14 (95% *CI*: 0.94–1.39), and 1.37 (95% *CI*: 1.11–1.69) for individuals in Q2, Q3, and Q4, respectively (*p*-trend < 0.01; basic model). Additionally, per 100 μmol/L of SUA level increase was significantly associated with 1.21 (95% *CI*: 1.09–1.36)-fold higher incident risk of diabetes in the basic model. Additionally, those participants diagnosed with hyperuricemia at baseline had a 41% increased risk of developing diabetes during follow-up (95% *CI*: 7–86%; *p* < 0.01). The observed association attenuated and was no longer significant after further adjustment for baseline BMI levels (basic model + BMI) or baseline dyslipidemia levels (basic model + dyslipidemia), whether for SUA as a continuous or categorical variable.

**Table 2:** Risk ratios (95% confidence intervals) of diabetes according to SUA.

	**Univariate**	**Basic model**	**Basic model + BMI**	**Basic model + Dyslipidemia**
	***RR* (95% *CI*)**	***RR* (95% *CI*)**	***RR* (95% *CI*)**	***RR* (95% *CI*)**
**Total participants**				
Q1	Ref.	Ref.	Ref.	Ref.
Q2	1.01 (0.83, 1.23)	1.00 (0.82, 1.23)	0.96 (0.79, 1.18)	0.99 (0.81, 1.21)
Q3	1.16 (0.95, 1.40)	1.14 (0.94, 1.39)	1.08 (0.89, 1.31)	1.10 (0.90, 1.34)
Q4	1.43 (1.18, 1.72)***	1.37 (1.11, 1.69)**	1.23 (0.99, 1.52)	1.28 (1.04, 1.57)*
*P* for trend^a^	<0.001	<0.01	0.11	0.01
Normal uric acid	Ref.	Ref.	Ref.	
Hyperuricemia	1.50 (1.16, 1.93)**	1.41 (1.07, 1.86)*	1.32 (0.99, 1.74)	1.33 (1.01, 1.75)*
Uric acid (100 umol/l)	1.17 (1.08, 1.27)***	1.21 (1.09, 1.36)**	1.15 (0.03, 1.29)*	1.17 (1.05, 1.30)*
**Male participants**				
Q1	Ref.	Ref.	Ref.	Ref.
Q2	1.00 (0.76, 1.32)	1.01 (0.77, 1.34)	0.97 (0.74, 1.29)	1.00 (0.76, 1.32)
Q3	0.86 (0.64, 1.15)	0.88 (0.655, 1.18)	0.84 (0.63, 1.13)	0.86 (0.64, 1.16)
Q4	1.05 (0.79, 1.38)	1.05 (0.77, 1.43)	0.98 (0.72, 1.34)	1.02 (0.75, 1.38)
*P* for trend^a^	0.882	0.559	0.748	0.729
Normal uric acid	Ref.	Ref.	Ref.	Ref.
Hyperuricemia	1.43 (1.01, 2.04)*	1.42 (0.99, 2.01)	1.32 (0.99, 1.74)	1.41 (0.97, 2.16)
Uric acid (100 umol/l)	1.05 (0.91, 1.21)	1.07 (0.90, 1.26)	1.04 (0.88, 1.23)	1.05 (0.89, 1.24)
**Female participants**				
Q1	Ref.	Ref.	Ref.	Ref.
Q2	1.09 (0.82, 1.45)	1.08 (0.81, 1.44)	1.05 (0.79, 1.40)	1.08 (0.81, 1.44)
Q3	1.40 (1.07, 1.83)*	1.34 (1.02, 1.77)*	1.25 (0.95, 1.65)	1.27 (0.97, 1.67)
Q4	1.81 (1.40, 2.34)***	1.64 (1.24, 2.18)**	1.45 (1.09, 1.93)*	1.49 (1.13, 1.97)*
*P* for trend^a^	<0.001	0.001	0.006	0.001
Normal uric acid	Ref.	Ref.	Ref.	Ref.
Hyperuricemia	1.61 (1.11, 2.35)*	1.36 (0.91, 2.03)	1.23 (0.81, 1.85)	1.27 (0.86, 1.88)
Uric acid (100 umol/l)	1.46 (1.29, 1.65)***	1.40 (1.21, 1.62)***	1.31 (1.12, 1.52)*	1.32 (1.15, 1.53)**

*p < 0.05.

**p < 0.01.

***p < 0.001.

*The based model was adjusted for age (standardized), gender, education levels, marital status and place of residence, high-sensitivity C-reactive protein, blood urea nitrogen, smoking status, drinking status, and hypertension.*

*^a^Test for linear trend was performed using the median SUA levels for each quartile as a continuous variable.*

*SUA, serum uric acid; Q1, Q2, Q3, and Q4 were the quartiles of SUA; BMI, body mass index*.

Stratified analysis by sex found that SUA was not significantly associated with the risk of diabetes in the men subgroup. Whereas, in women, the basic model is similar to the total population, compared with the first sex-specific quartile of SUA levels, the *RR*s of diabetes were 1.08 (95% *CI*: 0.81–1.44), 1.34 (95% *CI*: 1.02–1.77), 1.64 (95% *CI*: 1.24–2.18) for quartile 2–4 (*p*-trend = 0.001, basic model), respectively. A 100 μmol/L increment of SUA was linked with 40% (95% *CI*: 21–62%) elevated risk of diabetes in women (basic model). The observed association decreased but remained significant after further adjustment for BMI or baseline dyslipidemia levels in women (basic model + BMI: *p*-trend < 0.01; basic model + dyslipidemia: *p*-trend < 0.01). For women, we further stratified by menopause status. As shown in Table 3, after adjusting for confounders, the relation of baseline SUA and the risk of diabetes in premenopausal women were not statistically significant. In the postmenopausal female subgroup, the results were similar to the overall analysis of all female patients, no matter took uric acid as a continuous variable or dichotomic variable, and the increase of uric acid can lead to the increased risk of diabetes in postmenopausal women.

**Table 3:** Risk ratios (95% confidence intervals) of diabetes according to SUA in women subgroup analysis.

	**Univariate**	**Basic model**	**Basic model + BMI**	**Basic model + Dyslipidemia**
	***RR* (95% *CI*)**	***RR* (95% *CI*)**	***RR* (95% *CI*)**	***RR* (95% *CI*)**
**Without menopause (*****N*** **=** **1,545)**				
Q1	Ref.	Ref.	Ref.	Ref.
Q2	1.16 (0.67, 2.03)	1.16 (0.66, 2.03)	1.14 (0.65, 2.00)	1.15 (0.66, 2.02)
Q3	1.51 (0.88, 2.58)	1.40 (0.80, 2.44)	1.35 (0.78, 2.33)	1.31 (0.75, 2.29)
Q4	2.20 (1.34, 3.61)**	1.44 (0.83, 2.50)	1.30 (0.75, 2.62)	1.28 (0.73, 2.22)*
*P* for trend^a^	<0.01	0.557	0.698	0.700
Normal uric acid	Ref.	Ref.	Ref.	Ref.
Hyperuricemia	1.66 (0.71, 3.85)	1.38 (0.56, 3.41)	1.29 (0.52, 3.20)	1.27 (0.51, 3.15)
Uric acid (100 umol/l)	1.57 (1.25, 1.99)***	1.25 (0.95, 1.65)	1.18 (0.89, 1.57)	1.16 (0.88, 1.54)
**Menopause (*****N*** **=** **3, 290)**				
Q1	Ref.	Ref.	Ref.	Ref.
Q2	1.05 (0.75, 1.47)	1.04 (0.74, 1.47)	1.00 (0.72, 1.42)	1.05 (0.75, 1.48)
Q3	1.31 (0.96, 1.79)	1.28 (0.93, 1.76)	1.18 (0.85, 1.62)	1.23 (0.89, 1.69)
Q4	1.64 (1.22, 2.21)**	1.57 (1.15, 2.16)**	1.39 (1.01, 1.93)*	1.44 (1.05, 1.97)*
*P* for trend^a^	<0.01	0.02	0.02	0.04
Normal uric acid	Ref.	Ref.	Ref.	Ref.
Hyperuricemia	1.55 (1.03, 2.37)*	1.21 (0.76, 1.92)	1.08 (0.67, 1.75)	1.11 (0.71, 1.73)
Uric acid (100 umol/l)	1.40 (1.21, 1.62)***	1.35 (1.15, 1.58)***	1.26 (1.07, 1.49)**	1.28 (1.10, 1.49)**

*p < 0.05.

**p < 0.01.

***p < 0.001.

*The based model was adjusted for age (standardized), gender, education levels, marital status and place of residence, high-sensitivity C-reactive protein, blood urea nitrogen, smoking status, drinking status, and hypertension.*

*^a^Test for linear trend was performed using the median SUA levels for each quartile as a continuous variable.*

*SUA, serum uric acid; Q1, Q2, Q3, Q4 were the quartiles of SUA; BMI, body mass index*.

### The Crosslagged Path Analysis of Uric Acid and Blood Glucose

**Figure 3:**
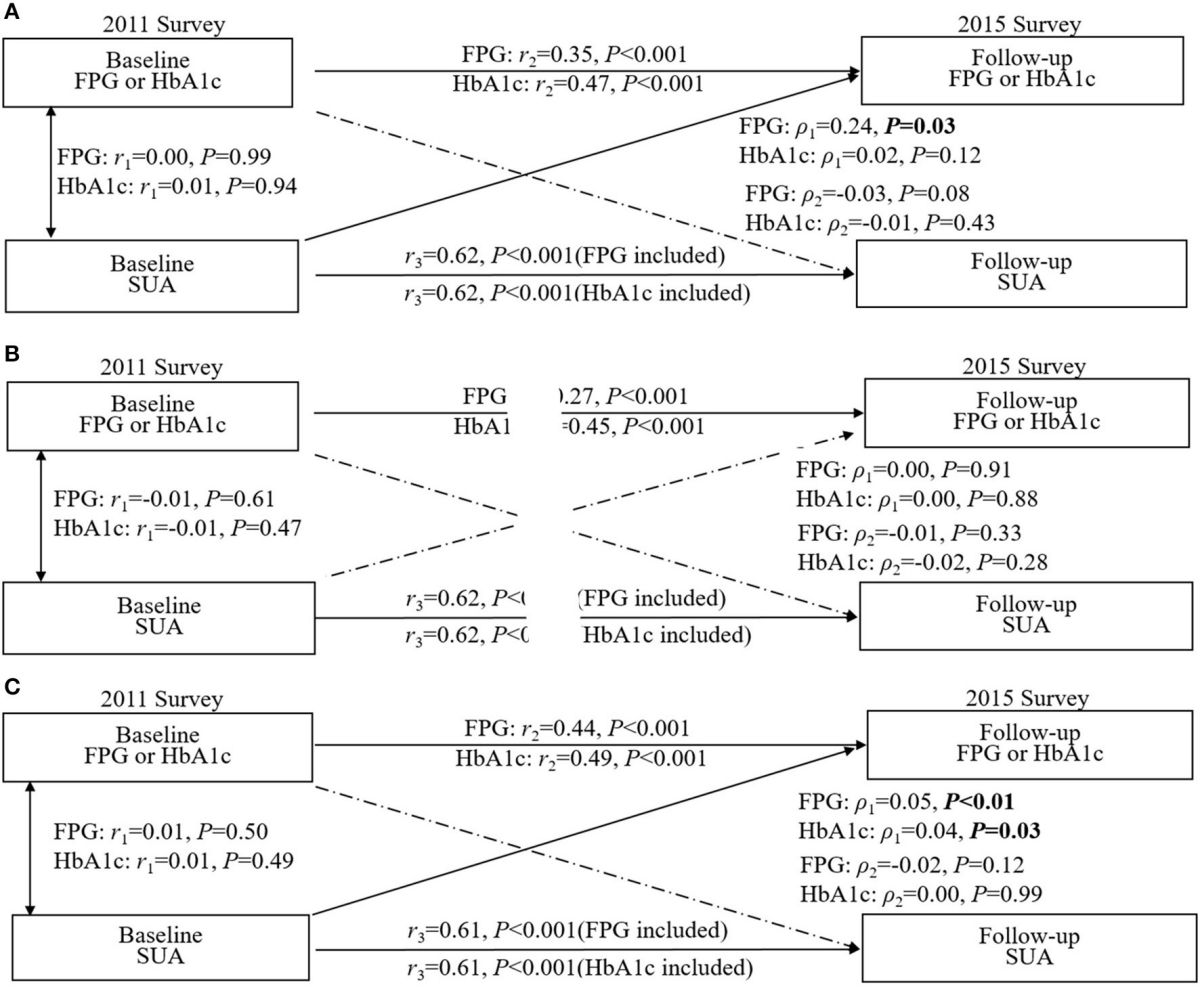
Crosslagged path analysis models for the association of fasting plasma glucose (FPG) and HbA1c with SUA. **(A)** included all participants (*n* = 6,873); **(B)** included male participants (*n* = 3,162); **(C)** included female participants (*n* = 3,711). All results were adjusted for age, sex, marital status, education background, waist circumference, smoking, drinking, hypertension and high-sensitivity C-reactive protein. ρ_1_, crosslagged path coefficient from baseline SUA to follow-up blood glucose (FPG and HbA1C); ρ_2_, crosslagged path coefficient from baseline blood glucose (FPG and HbA1C) to follow-up SUA. *r*_1_, represents synchronous correlations; *r*_2_ and *r*_3_ represent tracking correlations.

As shown in Figure 3A, in all pathways, only the path coefficients (ρ_1_) from baseline SUA to follow-up blood glucose (ρ_1_ = 0.24, *p* = 0.03) were significant in the total population after adjusting for confounding factors, and the RMR and CFI were 0.009 and 0.998 for FPG, 0.002 and 0.999 for HbA1c, respectively, indicating a good fit to the observed data, suggesting that SUA was more likely to affect blood glucose and is a risk factor for diabetes. Further stratified by sex, we found that this path coefficient between baseline uric acid and follow-up blood glucose was significant only in the female subgroup (for FPG: ρ_1_ = 0.05, *p* < 0.01; for HbA1c: ρ_1_ = 0.04, *p* = 0.03; Figure 3C), and no longer had a statistical association in men (Figure 3B).

### The Mediation Analysis in the Women Subgroup

**Figure 4:**
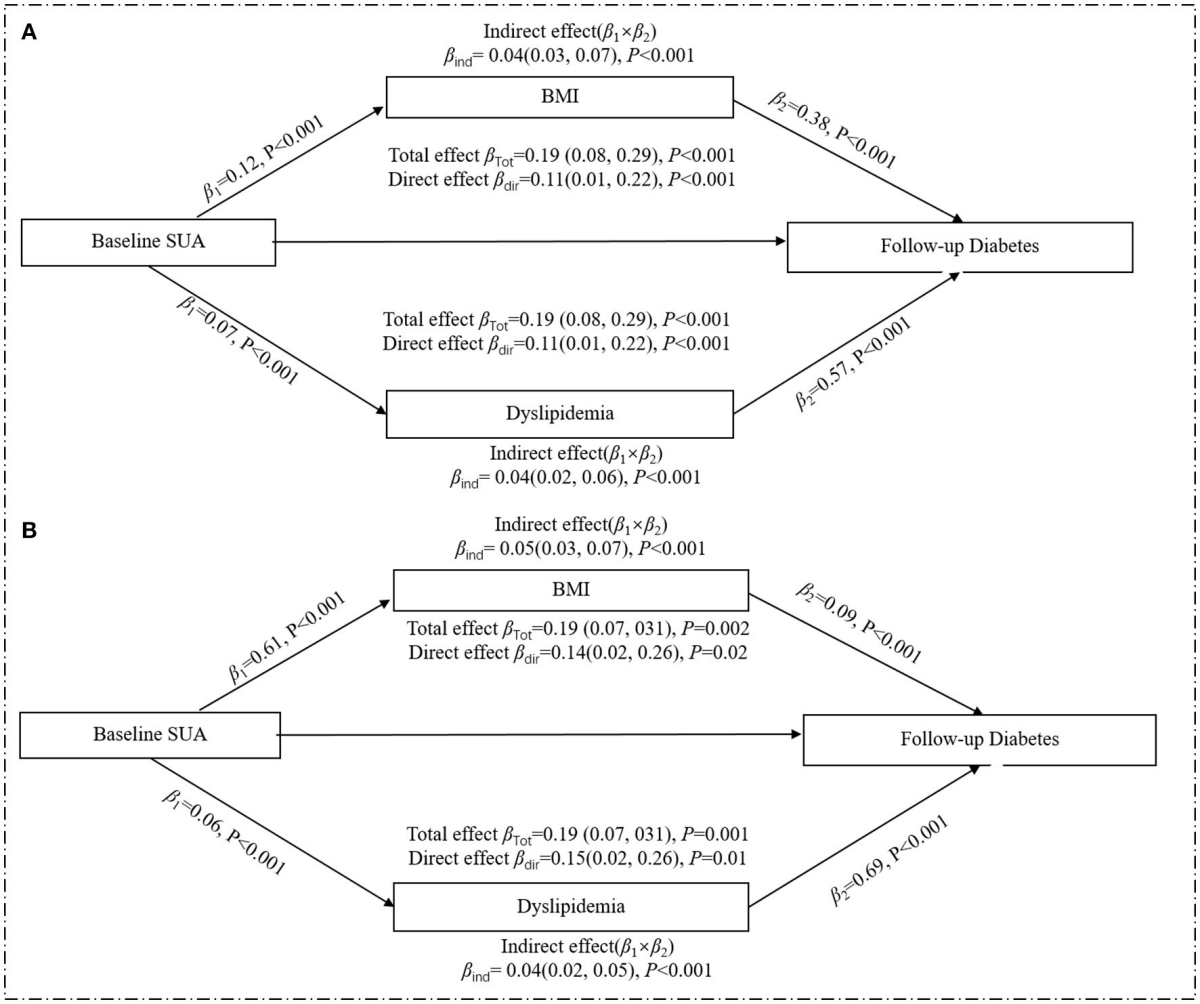
Mediation effect of baseline obesity on the relation of baseline SUA and risk of diabetes in the women. **(A)** included all women (*n* = 4,835); **(B)** included the postmenopausal female subgroup (*n* = 3,290). All analyses were adjusted age, marital status, education background, smoking, drinking, high-sensitivity C-reactive protein, menopausal status, and eGFR. The total effect is the effect of SUA on diabetes without considering BMI or dyslipidemia; the direct effect is the effect of SUA on diabetes when controlling for BMI and dyslipidemia; the indirect effect is the effect of SUA on diabetes through BMI or dyslipidemia. Mediation effects by BMI or dyslipidemia are calculated by indirect effect/total effect ×100. β, regression coefficients.

Since the above statistical analysis confirmed that SUA was an independent risk factor for the development of diabetes in women in terms of both the strength of association (*RR*) and the temporal sequence (crosslagged path analysis), we analyzed the mediating effects of BMI and dyslipidemia in the female subgroup, respectively (Figure 4). After adjusting for all potential confounding factors in Figure 4A, the increased level of baseline SUA was positively correlated with baseline BMI (β_1_ = 0.12, *p* < 0.001) or dyslipidemia (β_1_ = 0.07, *p* < 0.001), and the change in baseline BMI or dyslipidemia could significantly increase the incidence of diabetes (for BMI: β_2_ = 0.38, *p* < 0.001; for dyslipidemia: β_2_ = 0.57, *p* < 0.001). Similar results were found in a subgroup of postmenopausal women who were assessed separately for BMI and dyslipidemia (Figure 4B). Multiple parallel mediation analyses (Table 4) showed a significant total effect of baseline SUA on diabetes risk (β = 0.19, 95% *CI*: 0.08–0.29, *p* < 0.001), the direct effect of SUA on the relation of diabetes accounted for 58.13% (β = 0.11, 95% *CI*: 0.01–0.22, *p* < 0.001) of the total effect, and the combined indirect effect of BMI and dyslipidemia explained residual 41.78% (β = 0.8, 95% *CI*: 0.06–0.10, *p* < 0.001) of the total effect. Additionally, in the stratified analysis, we confirmed this mediating effect in postmenopausal women.

**Table 4:** Multiple parallel mediation effect of baseline BMI and dyslipidemia on the relation of baseline SUA and risk of diabetes in women.

	**Mediators**	**Effect**	**SE**	**95% *CI***	***P***	**Proportion**
**Total women (*****N*** **=** **4,835)**						
Direct effect		0.11	0.05	(0.01, 0.22)	<0.001	58.13%
Indirect effect	BMI+ Dyslipidemia	0.08	0.01	(0.06, 0.10)	<0.001	41.87%
	BMI	0.04	0.01	(0.03, 0.07)	<0.001	23.05%
	Dyslipidemia	0.04	0.01	(0.02, 0.06)	<0.001	18.82%
Total effect		0.19	0.05	(0.08, 0.29)	<0.001	100%
**Postmenopausal women (*****N*** **=** **3,290)**						
Direct effect		0.12	0.06	(−0.01, 0.23)	0.06	60.68%
Indirect effect	BMI+ Dyslipidemia	0.07	0.01	(0.05, 0.10)	<0.001	39.32%
	BMI	0.04	0.01	(0.03, 0.07)	<0.001	23.05%
	Dyslipidemia	0.03	0.01	(0.02, 0.06)	<0.001	18.82%
Total effect		0.19	0.06	(0.07, 0.31)	<0.001	100%

*The mediation model is based on the Karlson–Holm–Breen (KHB) method. All analysis was adjusted age, marital status, education background, smoking, drinking, high-sensitivity C-reactive protein, and blood urea nitrogen.*

*The total effect is the effect of SUA on diabetes without considering BMI or dyslipidemia; the direct effect is the effect of SUA on diabetes when controlling for BMI and dyslipidemia; the indirect effect is the effect of SUA on diabetes through BMI or dyslipidemia; proportion: mediation effect by BMI or dyslipidemia is calculated by indirect effect/total effect ×100*.

## Discussion

This study represents a comprehensive examination of the longitudinal relationship between uric acid concentration and diabetes in a national-based population. Our results help reconcile conflicting evidence in the literature and demonstrate the following: (1) an unidirectional relationship between SUA and blood glucose was identified, and increased SUA is an independent risk factor for diabetes; (2) In men, the longitudinal association between SUA and diabetes was not significant after adjustment for confounders.; (3) In women, SUA is an important risk factor for the development of diabetes, especially in postmenopausal women, and this harmful effect of uric acid is partly mediated by BMI and dyslipidemia.

Since the sex-specific cutoff points for hyperuricemia have been widely used globally, it is of importance to identify whether there are sex-specific associations between SUA and diabetes. Previous studies reported that SUA increment was associated with increased risk for diabetes in women but not in men (32, 33). Consistent with this, we confirmed that for every 100 μmol/L increase in SUA in women, the risk of diabetes was 1.31-fold higher in women. Not only in its association with diabetes but also gender have differences also often been seen in other fields. In the Framingham Heart Study, levels of SUA were associated with an increased risk of cardiovascular death in women but not in men (34). Additionally, Ndrepepa et al. also demonstrated that hyperuricemia could predict an increased cardiovascular risk of mortality in both genders, with a stronger association in women (35). Possible mechanisms for gender differences include the following: first, there were differences in BMI, hypertension, WC, BUN, eGFR, and blood lipid between genders, which partly explained the different reactivities of uric acid and blood glucose in men and women; second, a reduction in estrogen levels after menopause in women may result in dysregulation of blood glucose and lipid metabolism (36), and of the 4,835 women included in this study, about 68% were postmenopausal, and our subgroup analysis proved that elevated uric acid increased the risk of diabetes only in postmenopausal women. Besides, a genome-wide association study reported a significant association between the SLC2A9 and urate concentrations, whereas the proportion of the variance of SUA concentrations explained by expression levels was 3.5% in men and 15% in women (37), which possibly suggests a genetic basis for the sex differences.

The mediating effect is based on the premise that the causal relationship between independent and dependent variables holds. Previous studies have identified uric acid as a risk factor for diabetes (18, 19, 38); however, Lu et al. used an animal model which confirmed that hyperuricemia could accelerate but do not cause diabetes by inhibiting islet β-cell survival (39); only male mice were used in their study, which may impede the extrapolation from animal experiments to human populations. Meanwhile, several Mendelian randomized (40–42) studies also do not support a causal role of SUA for the development of diabetes and limit the expectation that UA-lowering drugs will be effective in the prevention of diabetes (39); whereas the genetic risk score in above Mendelian randomization studies only explained 2.9% of SUA variation, the strength of the evidence may be insufficient. Taking the above results into consideration, we further explored this chicken-and-egg question by crosslagged path analysis, which is a powerful statistical approach in dissecting a causal relationship between intercorrelated variables (29). In our results, the path coefficient from baseline SUA to follow-up blood glucose was statistically significant in the total population and the female subgroup, providing statistical evidence that uric acid is a possible cause of blood glucose; therefore, we only explore the potential mediation effect in women.

Similar to our findings, some research pointed out that the association between SUA and the risk of diabetes is largely decreased or eliminated after adjusting for BMI (10, 11). UA can affect adipocytes by inducing upregulation of proinflammatory factors and downregulation of the insulin sensitizer and antiinflammatory factor adiponectin (43). Adiponectin is negatively associated with BMI and body fat (44) since low levels of adiponectin are associated with the development of insulin resistance (45), and it could be speculated that adiponectin is part of the link between UA and insulin resistance (46). Additionally, data coming from the National Health and Nutrition Examination Survey (NHANES) demonstrated a significant association between elevated SUA levels and the increased prevalence of abdominal obesity, hypertriglyceridemia, and hyperglycemia (47), and some prospective studies also showed that elevated SUA levels may increase the risk of hypertriglyceridemia (48). Hypertriglyceridemia is known as a dominant lipid abnormality in insulin resistance by inducing elevated levels of free fatty acids, which plays an important role in the development of diabetes (49). The interactive effects of increased TG and the LDL-C/HDL-C ratio suggest that dyslipidemia might exaggerate the risk of diabetes in hypertensive patients (50). This study demonstrated that nearly 42% total effect of UA on diabetes is mediated by BMI and dyslipidemia in women. This provides a theoretical basis for the designation of preventive measures. For middle-aged and older women, especially those with higher uric acid levels, reasonable diet control and physical exercise can keep them in the normal BMI range and reduce the probability of dyslipidemia, which can help us avoid developing diabetes in the future.

Strengths of this study include a wealth of sociodemographic, clinical, and laboratory variables collected by medical professionals in a standardized manner with a low rate of missing data. In addition, the confirmation of multiple mediating roles of BMI and dyslipidemia helps to clarify the pathogenetic pathway of uric acid to diabetes. What is more, CHARLS adopts probability proportional to size sampling, so the results can represent the current level of China and have good extrapolation. Study weaknesses include our follow-up period which was only 4 years, which was comparatively shorter than other cohort studies, and despite adjustment for a range of potential confounders, the possibility of residual and unmeasured confounders may not be ruled out, such as diet, drugs, and genetic information. Besides, as in any observation study, causality cannot be determined by the strength of relation and temporal relation alone, and more definitive basic studies are warranted to confirm causality between UA and diabetes.

## Conclusion

In conclusion, using a sample drawn from the China Health and Retirement Longitudinal Study, this study extended the findings of the previous literature by confirming that SUA and risk of type 2 diabetes are only significant in middle-aged and elderly Chinese women, and further quantified the mediating proportion of BMI and dyslipidemia in the relationship between SUA and risk of type 2 diabetes in women. For middle-aged and elderly Chinese women, especially those with high uric acid, in addition to corresponding measures to reduce uric acid, integrally targeted interventions and strategies that can alleviate BMI and dyslipidemia should be combined to reduce the risk of diabetes.

## Data Availability Statement

The datasets presented in this study can be found in online repositories. The names of the repository/repositories and accession number(s) can be found at: the CHARLS data can be freely downloaded from the official website (http://charls.pku.edu.cn/index/zh-cn.html).

## Ethics Statement

The studies involving human participants were reviewed and approved by the CHARLS is a large-scale interdisciplinary research project sponsored by the National Development Institute of Peking University. Ethical approval for all the CHARLS waves, therefore, was granted from the Institutional Review Board (IRB) at Peking University, including anthropometrics (IRB00001052-11015) and biomarker collection (IRB00001052-11014). The patients/participants provided their written informed consent to participate in this study.

## Author Contributions

HJ designed the study. FC performed the statistical analyses, drafted the manuscript, and created the figures. YL, HZ, and LT interpreted the data and edited the manuscript. All authors listed have approved the manuscript that is enclosed and the corresponding author would take final responsibility for the paper.

## Funding

This project was supported by the Research Development Fund of The Second Hospital of Shandong University (Grant No. 11681808), Construction of Intelligent Cloud Platform for Clinical Medicine Teaching based on multi-disciplinary typical cases (Grant No. 2019Z10), and Jinan Clinical Medical Science and Technology innovation plan (Grant No. 202019194).

## Conflict of Interest

The authors declare that the research was conducted in the absence of any commercial or financial relationships that could be construed as a potential conflict of interest.

## Publisher's Note

All claims expressed in this article are solely those of the authors and do not necessarily represent those of their affiliated organizations, or those of the publisher, the editors and the reviewers. Any product that may be evaluated in this article, or claim that may be made by its manufacturer, is not guaranteed or endorsed by the publisher.
